# Maintaining long-term physical activity after cancer: a conceptual framework to inform intervention development

**DOI:** 10.1007/s11764-023-01434-w

**Published:** 2023-08-14

**Authors:** Chloe Grimmett, Teresa Corbett, Katherine Bradbury, Kate Morton, Carl R. May, Bernardine M. Pinto, Claire Foster

**Affiliations:** 1 Centre for Psychosocial Research in Cancer: CentRIC+, School of Health Sciences, University of Southampton, Southampton, UK; 2 Faculty of Sport, Health and Social Sciences, Solent University, Southampton, UK; 3 School of Psychology, University of Southampton, Southampton, UK; 4 Faculty of Public Health and Policy, London School of Hygiene and Tropical Medicine, London, UK; 5 College of Nursing, University of South Carolina, Columbia, SC USA

**Keywords:** Cancer, Physical activity, Maintenance, Conceptual framework, Intervention

## Abstract

**Purpose:**

This paper describes a conceptual framework of maintenance of physical activity (PA) and its application to future intervention design.

**Methods:**

Evidence from systematic literature reviews and in-depth (*N* = 27) qualitative interviews with individuals with cancer were used to develop a conceptual framework of long-term physical activity behaviour. Determinants of long-term PA were listed and linked with domains of the Theoretical Domains Framework which in turn were linked to associated behaviour change techniques (BCTs) and finally to proposed mechanisms of action (MoA).

**Results:**

The conceptual framework is presented within the context of non-modifiable contextual factors (such as demographic and material resources) and in the presence of learnt and adapted behavioural determinants of skills, competence and autonomous motivation that must be established as part of the initiation of physical activity behaviour. An inventory of 8 determinants of engagement in long-term PA after cancer was developed. Clusters of BCTs are presented along with proposed MoA which can be tested using mediation analysis in future trials.

**Conclusion:**

Understanding the processes of PA maintenance after cancer and presentation of implementable and testable intervention components and mechanisms of action to promote continued PA can inform future intervention development.

**Implications for Cancer Survivors:**

This resource can act as a starting point for selection of intervention components for those developing future interventions. This will facilitate effective support of individuals affected by cancer to maintain PA for the long term.

## Introduction

Each year, more than 17 million people are diagnosed with cancer worldwide [1] . Cancer and its treatment can result in numerous adverse physical and psychological consequences, some of which can persist for years after treatment completion [2]. Individuals with a history of cancer are also at risk of cancer recurrence and developing other chronic conditions such as heart disease. Engaging in regular physical activity (PA) can mitigate many of these adverse effects including fatigue, anxiety, depression, physical functioning and health-related quality of life [3] and reduce cancer recurrence and improve survival [4].

Despite this, around 70% of people with cancer are not meeting physical activity guidelines [5–8]. Systematic reviews and meta-analysis have found clinically significant effects of interventions to support initiation of physical activity behaviour change with substantial increases in activity levels from baseline to end of intervention [9–11]. However, to sustain the health benefits of physical activity, individuals must be habitually physically active for the long term. There is some evidence of modest maintenance effects [12] in people with cancer but activity levels typically regress towards baseline as the time from end of intervention increases [13]. There are scant examples of interventions developed with an explicit aim to support sustained increases in physical activity in people affected by cancer and further research is required.

Key to developing effective interventions is to identify determinants of the target behaviour in the population in question as well as potential processes of change. Intervention components that influence those determinants can then be identified. This can be achieved by reviewing existing literature and consultation with the intervention development team. It is also increasingly recognised that empirical research with the specific population is imperative to fully understand the ‘problem’ and identify potential solutions. These are core principles of intervention development frameworks such as Intervention Mapping and the MRC Guidance for development of complex interventions [14]*.*

Such development frameworks also recommend the use of theory of behaviour/behaviour change to guide the identification of pathways of change and appropriate intervention components. The theory of planned behaviour, social cognitive theory and the transtheoretical model have received much attention within the PA and cancer literature [15–17]. However empirical evidence suggests no one theory is superior and that theory-based interventions are as effective as those without explicit theoretical underpinning [18, 19]. This may be because both types of intervention include similar behaviour change techniques (the main catalysts of intervention effects) [14, 18] and existing theories have multiple overlapping constructs. To address the latter issue, the Theoretical Domains Framework (TDF) was developed. It brings together 33 models of behaviour/behaviour change, including 128 separate constructs, and is increasingly used by researchers developing complex interventions that target health behaviour change [20]. The TDF has 14 theoretical domains from which researchers can draw on to support identification of pathways of behaviour change.

Furthermore, for optimal transparency of the intervention development process, and to advance our understanding of not just *what* works, but *how* it works, researchers are encouraged to identify the proposed mechanisms of action (MoA) through which interventions are hypothesised to exert their effects. MoA are ‘the processes through which behaviour change techniques affect behaviour’ [21]. For example, a barrier to engagement in physical activity might be a lack of belief in one’s capability to perform the behaviour. Intervention developers would select BCTs believed to impact the MoA ‘belief in capability’, for example, verbal persuasion and focus on past success. Subsequent evaluation of the intervention would include measuring change in this MoA and conducting mediation analysis to determine its impact on behaviour [22]. Central to such endeavours is an agreed matrix of BCTs and hypothesised MoA which can act as a standardised resource, enabling synthesis of data. This has been achieved through a series of studies of literature synthesis [23] and expert consensus [24], triangulated to develop a Theory and Technique tool linking BCTs and their MoA [25].

To date, there is a lack of empirical data, conceptual or theoretical understanding of the process of engaging in sustained physical activity that can inform the identification of behavioural determinants. Indeed, in concluding remarks following their recent meta-analysis of sustaining physical activity behaviour after intervention completion, McEwan et al. (2021) recommend ‘future efforts to develop and test theoretical frameworks that specially focus on maintenance of health behaviour could help optimise interventions that are concerned with supporting long-term physical activity adherence’ (p8).

This paper describes the development of a conceptual framework of sustained physical activity engagement in people with cancer through meta-analysis and primary qualitative research. This led to the generation of an inventory of determinants of this behaviour and a corresponding matrix of BCTs and associated MoA that can be used as a basis for developing future interventions.

## Methods

A modified version of French et al.’s [26] approach to developing theory informed behaviour change interventions was used and involved four phases:

1. Who needs to do what, differently?
  A systematic review and meta-analysis of long-term physical activity behaviour change in cancer survivors was conducted by our group [12]. We explored factors associated with success (or lack thereof) including context, population characteristics and behaviour change techniques.
2. What barriers, enablers and processes need to be addressed?
  To afford an in-depth understanding of the barriers, enablers and processes involved in long-term PA behaviour change a qualitative study of 27 cancer patients who had taken part in a previous PA programme in the UK was conducted [27]. Inductive thematic analysis was used, and findings were combined with those from the aforementioned systematic review and meta-analysis [15] as well as evidence from other relevant qualitative meta-syntheses and reviews [13, 28–30]. Factors were selected and structured to create constructs that informed the development of an inventory of key determinants of sustained PA behaviour. These data also informed the development of a conceptual framework describing the processes of sustained PA engagement.
3. Using a theoretical framework, what intervention components could address these barriers and enablers?
  In consultation with co-authors, barriers and enablers were mapped to the associated domains of the Theoretical Domains Framework. Using published expert consensus linking BCTs to the TDF domains [31], BCTs were identified that might address these barriers and enablers. In addition, we reviewed the recently published compendium of ‘self-enacted techniques’ [32] and selected additional intervention components hypothesised to impact on the target determinants.
4. How can the behaviour change be understood?
  Using the Theory and Techniques Tool, we identified key MoA associated with each behaviour change technique which could be used to assess intervention causal pathways (mediating behaviour change).
  This was an iterative process involving regular meetings and revisions at all stages with co-authors.

## Results

### Who needs to do what, differently?

The systematic review and meta-analysis, including 19 studies, concluded that existing interventions with a long-term follow-up were successful in achieving moderate improvements in sustained behaviour change [12]. Older adults, those with existing functional limitations and who had fewer contacts with those delivering the PA programme were less likely to sustain PA increases. Furthermore, PA programmes included in the review with poorer long-term behaviour outcomes were less likely to include the BCTs of action planning, graded tasks and social support (unspecified) (see [12] for full details). These findings were triangulated with the qualitative data [27] and relevant reviews [13, 28–30] generating hypotheses of the processes at play and informing step 2: generation of an inventory of barriers and enablers and conceptual framework.

### What barriers, enablers and processes need to be addressed?

**Fig. 1:**
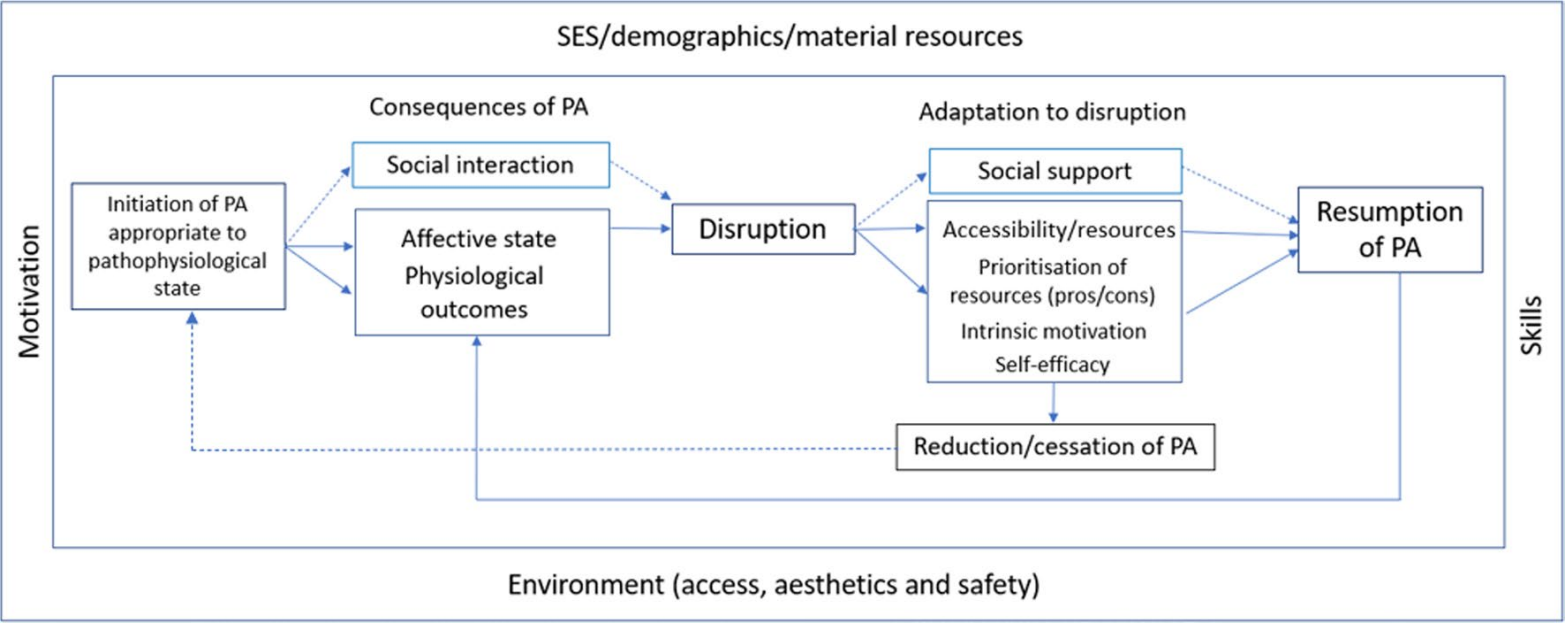
Long-term PA behaviour after cancer

Findings from the qualitative study [27] and systematic reviews [12, 18, 28–30] were selected and structured to identify an inventory of 8 determinants of engagement in long-term PA behaviour after cancer and a conceptual framework illustrating the processes at play was developed (see Fig. 1).

Central to the conceptual framework are the founding factors related to initiation of behaviour required before long-term maintenance can be achieved (depicted in the outer segment of Fig. 1). Such factors are vital for consideration during the initiation of PA behaviour before maintenance can be achieved. Contextual factors are key and include socioeconomic status, demographics (including age, sex, education level), material resources and environment (i.e. access to facilities and/or appealing outdoor space). Appropriate attention must be paid to these factors when identifying when, where and how an individual will engage in a physically active behaviour. This will ensure the chosen activity is appropriate to the individual’s personal context and resources. Furthermore, the initial intervention must result in *motivation* to initiate change. The participants must develop the necessary *skills* to engage with their new activity and that activity must be appropriate to their pathophysiological status.

*A case study: A participant in the qualitative study described how the practice nurse at her GP surgery repeatedly told her she needed to do more exercise and suggested walking. No support or consultation was provided. The participant reported walking on a handful of occasions but then stopped. Reasons included pain on walking, concerns about breathlessness and difficulties accessing suitable walking routes. On taking part in the Move More intervention, she engaged in a conversation with the practitioner regarding her history of PA, likes and dislikes, her priorities and commitments as well as existing health condition, which included obesity, mobility issues/joint pain and COPD. A local weekly yoga class was identified which, on the date of interview, the participant had been attending for more than 2 years.*

Once PA behaviour is initiated, the individual experiences the consequences of that behaviour, including an impact on their affective state and physiological outcomes. When engaging in PA with others, this will also include social interaction. If the behaviour results in desired/positive outcomes, that behaviour is reinforced and is more likely to be sustained. In most cases, at some point, the behaviour will be disrupted. This may be due to affective factors, such as low mood or boredom, or practical, e.g. discontinuation of an exercise class. Alternatively, a life stressor event such as ill health or caring responsibilities can disrupt engagement, as can change to the environment/resources. Adaptation to this disruption then ensues. This will include a prioritisation process of physical and psychological resources. It may also involve problem solving, assessment and/or change regarding accessibility to local amenities and/or personal resources. Pros and cons of re-engaging with the discontinued activity including reflection on the consequences of behaviour (experienced outcomes) will be influenced by the degree of intrinsic motivation and self-efficacy (confidence) the individual has for that activity. For some, social support will play a role here with some individuals requiring practical or emotional support to re-engage. Consequently, the behaviour remains ceased/reduced or resumption occurs. This is typically a cyclical process.

The inventory of barriers and enablers are set out in Table 1.

**Table 1:** Inventory of behaviour change techniques and mechanisms of action

Determinants of engagement	Subcategories of taxonomy	TDF domain	BCT linked to TDF domain	MoA linked to BCT	Self-enacted techniques
Enjoyment/pleasure		Emotion	11.2 Reduce negative emotions	Emotion; behavioural regulation	#19 Monitoring of emotional consequences #29 Task crafting (enjoyment) #109 Focus on enjoyment (pleasant aspects) of behaviour #119 interpreting physiological and emotional states #98 Reflect on desire to perform behaviour #100 Reflect on reasons to perform the behaviour
			5.4 Monitoring of emotional consequences		
			3.3 Social support (emotional)		
Exercise promoting feelings of empowerment		Belief about capability	15.1 Verbal persuasion about capability	Belief about capability	#107 Verbal self-persuasion about own capability #106 Valued self-identity (personal strengths) #111 Focus on past success #112 Looking back
			15.3 Focus on past success	Belief about capabilityMotivation	
			15.4 Self-talk		
			5.4 Monitoring of emotional consequences		
Perceived value/outcome	Extrinsic outcomes to include maintaining health/function and independence	Belief about consequences	5.2. Salience of consequences	Belief about consequences; perceived susceptibility/vulnerability	#42 Contrast/compare pros and cons #40 Make consequences more memorable #32 Goal integration—modify behaviour to simultaneously engage in another valued behaviour #104 Incompatible beliefs #102 Find meaning in target behaviour #105 Associate identity with changed behaviour
	Intrinsic outcomes (e.g. feelings of wellbeing)		5.5 Anticipated regret	Belief about consequences	
			9.3 Comparative imagining of future outcomes	Belief about consequences	
			16.3 Vicarious consequences		
			9.2 Pros and cons	Belief about consequences; attitude towards behaviour; motivation; general attitude/belief	
Exercise as a means of social interaction		Environmental context	7.1 Prompts/cues	Memory, attention and decision process; environmental context and resources; behavioural cueing	#54 Obtain practical social support
			12.2 Restructuring social environment	Environmental context and resources	
Disruption	Affective (boredom, anxiety, low mood)	Emotion	11.2 Reduce negative emotions 5.4 monitoring of emotional consequences 3.3 Social support (emotional)	Emotion; behavioural regulation	#19 Monitoring of emotional consequences #29 Task crafting (enjoyment) #109 Focus on enjoyment (pleasant aspects) of behaviour #119 Interpreting physiological and emotional states #31 Add challenge (stops behaviour becoming boring) #102 Find meaning in target behaviour #32 Goal integration #114 Normalise difficulty #115 Self-kindness #manage negative emotions
	External disruption (exercise class cancelled)	Environmental context	12.1 Restructuring the physical environment	Environmental Context and Resources; behavioural cueing	#25 Restructuring the physical environment #26 Restructuring social environment
			12.2 Restructuring the social environment	Environmental context and resources	
	Ill health as a disruption—cancer specific/comorbid conditions/minor illness	Environmental context	1.2 Problem solving	Belief about capability; behavioural regulation	
			1.4 Action planning	Behavioural cueing	
	Life stressors—i.e. caring responsibilities/work commitments	Environmental context	1.2 Problem solving	Belief about capability; behavioural regulation	
			1.4 Action planning	Behavioural cueing	
Perceived appropriateness for age		Social influence	6.2 Social comparison	Norms; subjective norms; feedback processes	#30 Task crafting (skills and ability)
			10.4 Social reward	Reinforcement; social influence	
Confidence/self-efficacy		Belief about capability	15.1 Verbal persuasion	Belief about capability	#112 Looking back
			15.3 Focus on past success	Belief about capability	
Monitoring	Self-monitoring	Behavioural regulation	2.3 Self monitoring of behaviour	Feedback processes; behavioural regulation	#17 Self-monitoring # reflect on desire to perform behaviour
	Monitoring by others	Social influence	2.2 Feedback on behaviour	Motivation; feedback processes	#15 Obtain feedback on behaviour #60 Prompts/cues #55 Obtain emotional support

Finding *pleasure*/*enjoyment* in the chosen activities was a key enabler of maintenance identified in the qualitative data and wider literature. Those individuals who were habitually active talked about the fun they had and enjoyment they felt from being physically active. Individuals engaging regularly in physical activity describe *feelings of empowerment* as a result, seeing it as evidence that they have overcome the physical challenges of cancer and its treatment and regaining a sense of ownership and control of their bodies. This is linked to a feeling of *confidence* in their ability to engage in these activities. An individual’s sense of *perceived value*/*experienced outcomes* is also integral to motivation to continue to engage in PA participation. Such outcomes included extrinsic factors such as the importance of PA in maintaining health, function and independence, as well as intrinsic factors such as a sense of wellbeing. For those who participate in group activities, *social interaction* is an important facilitator to engagement. *Disruption* to PA engagement can typically be divided into affective factors, such as low mood, anxiety and boredom and external factors including terminated exercise classes, ill health or stressors of daily life. The *perceived appropriateness* of PA to an individual’s age is also important with individuals less inclined to engage if social comparison or personal identity are not aligned with the activities. Finally, *monitoring* of engagement by self or others is an important element of participation. This process enables individuals to track progress and identify reductions in PA that can then be addressed.

### Using a theoretical framework, what intervention components could address these barriers and enablers?

To identify appropriate intervention components, the inventory of determinants was mapped to domains of the TDF and in turn to associated BCTs (per the Behaviour Change Techniques Taxonomy V1 [33]) as determined by published expert consensus [31]. Cane et al. (2015) did not include the full list of BCTs as per the V1. Those missing were reviewed and included if, through consensus, they were felt to be appropriate. Intervention components as specified in the Compendium of Self-Enacted Techniques [32] were also selected by identifying techniques that were theorised to influence the identified barriers and enablers and associated MoA. See Table 1 for details.

The TDF domains captured include emotion (positive/negative affect), environmental context and resources, social influences, beliefs about consequences, beliefs about capabilities and behavioural regulation. Accompanying BCT clusters (as per the BCT taxonomy) include natural consequences, antecedents, goals and planning, social support, self-belief, feedback and monitoring. Knittle et al.’s [32] compendium of self-enacted techniques has several overlapping techniques with Michie’s taxonomy but also includes some important additions which we hypothesise would impact some of the barriers and enablers identified here. For example, boredom was a barrier to continued PA engagement. This might be overcome with the use of the self-enactment technique ‘add challenge (stops behaviour becoming boring)’. Enjoyment was an important enabler to continued PA and the techniques of ‘task crafting (enjoyment)’ and ‘focus on enjoyment (pleasant aspects)’ are likely to facilitate this.

### How can the behaviour change be understood?

Mechanisms of action associated with the BCTs were identified using the Theory and Techniques Tool [34]. See Table 1. Measuring them in future interventions would enable calculation of mediating mechanisms of behaviour change.

## Discussion

This paper presents a novel conceptual model of long-term engagement in physical activity after a cancer diagnosis, with accompanying inventory of determinants and suggested behaviour change techniques. This resource can act as a starting point for selection of intervention components for those developing future interventions. It is essential that it is used alongside a robust development process, using qualitative and co-design approaches to understand the local context and specific participant group, working with multidisciplinary teams to choose methods and modes of delivery that are locally feasible.

This conceptual framework is a holistic appreciation of the problem of sustained PA participation after a cancer diagnosis. The outer elements of the framework, including contextual factors such as environment, socioeconomic status and material resources, are key, shaping the possibility of initial PA engagement which is necessary before sustained action can be achieved. Historically, consideration of these contextual factors has often been absent from intervention development. A personalised approach, assessing pathophysiological state and evaluating these contextual factors at an individual level, is key to establishing initial PA engagement that has the potential to be sustained. This perspective is in keeping with the recently updated MRC Framework for the Development and Evaluation of Complex Interventions [14], a key addition to which was the inclusion of context in the definition of complex interventions. It also included considerations for the systems within which the intervention is situated.

Comparisons can be drawn between the conceptual model, inventory of behavioural determinants and associated intervention components presented here and Kwasnicka et al.’s review of the theoretical explanations for maintenance of physical activity behaviour [35]. Kwasnicka and colleagues coded constructs of 100 theories of behaviour and set out five theoretical themes they deem relevant to PA maintenance, four of which are also captured in our work. These include ‘maintenance motives’; regular gratification is more likely to lead to sustained engagement, ‘self-regulation’, including the need for high coping self-efficacy, ‘resources’; psychological and physical assets; and environmental and social influences.

In recent years, the concept of behavioural maintenance in physical activity has received considerable attention with thought paid to the way maintenance is conceptualised and operationalised. The conceptual model presented here aligns with the evolution in thinking from maintenance of a behaviour as a specific state, to consideration of the underlying mechanisms of action that determine behaviour. This includes shifts back and forth between reflective and reactive processes [36]. Rhodes and Sui [36] present a new, working definition of physical activity maintenance as ‘a dynamic development of mechanisms of action that engender greater perceived behavioural enactment efficiency that partially supplant prior mechanisms of action that required greater perceived cognitive recourses to enact physical activity’. They argue that some constructs that were critical to the initiation of a behaviour will still be important for engagement in the longer-term. This is reflected in the conceptual model and associated BCTs presented here where constructs and techniques are essential to embed in the process of initial behaviour enactment to support longer term maintenance. Whilst it has been argued that maintained behaviours are characterised by intrinsic motivations and self-determined actions, self-regulatory skills are still required over time as inevitable disruptions occur and more effortful regulation is required. Hence, the suggested inclusion of BCTs that consolidate self-regulatory processes and skills in the initiation of behaviour change, as well as considering long-term behaviour change where these skills may need to be revisited to manage a period of disruption. Furthermore, some individuals may spend longer in a phase of effortful self-regulation than others and need to revisit these skills more frequently. This is also supported by evidence from a review of determinants of physical activity behaviour in older adults which found that self-regulatory strategies such as action planning and coping planning were positively associated with both activity initiation and maintenance [37]. Development of a typology of sustained physical activity that proceeded and informed this model argues that individuals fall into three ‘types’ [27]: those who, after initial support to engage, successfully maintain increased levels of PA through planning and prioritisation; those who are ‘intermittently active’ with cycles of action and inaction with frequent periods of effortful self-regulation; and those with consistently low levels of PA with minimal engagement in PA during or after intervention participation. Reviewing individuals after 12 weeks of intervention participation to ascertain which of these ‘types’ they most align with could help personalise the intervention components/BCTs that need to be emphasised to support maintenance of behaviour.

An important consideration when developing interventions including evidence-derived BCTs is to have confidence that these strategies are being delivered and utilised by recipients as intended. Knittle et al. [32] recently published the compendium of self-enactable techniques v1.0 to change and self-manage motivation and behaviour. The focus of this taxonomy is that of techniques which individuals can enact themselves, rather than those which are delivered or enacted by the intervention providers. They point to evidence that suggests maintenance of behaviour change following interventions is dependent on the extent to which individuals can enact the BCTs involved [38–40]. They also argue that existing taxonomies have insufficient focus on the way techniques are delivered, received (comprehended and understood) and enacted in everyday lives. This new classification also includes techniques from additional behavioural domains that may have utility in affecting behaviour and/or its determinants, from sport and occupational psychology. Finally, it is written in ‘plain, accessible language’ and includes ‘adequate instructions and examples to facilitate ease of use by practitioners and the general public’. In this paper, we have selected techniques that map to determinants of long-term PA engagement after cancer. Some have considerable overlap with those derived from Michie’s BCT taxonomy (but with accessible examples/definitions to optimise delivery and enactment) whereas others are unique and with intuitive application, for example, ‘task crafting (enjoyment)’ which specifically addresses the concept of making the target behaviour enjoyable, a behavioural determinant for long-term physical activity that is consistently reported in the literature but not explicitly addressed in previous taxonomies. Similarly, ‘normalise difficulty’ is defined as ‘recognise (or remind yourself) that is it common to face difficulties when pursuing behavioural changes’.

Consideration of how chosen BCTs are both delivered and received may help those developing and delivering interventions to maximise fidelity of both delivery and individual enactment. Examples of this include Miles et al. [41] who used qualitative methods to explore participant’s understanding of the specific BCTs included in the Diabetes Prevention Program. The focus was self-regulatory strategies such as problem-solving, action planning, self-monitoring of behaviour and goal setting. Some techniques, including self-monitoring, were well understood and participants accurately described their use. Others, including action planning and problem solving, were harder to understand and additional support was needed to enable participants to operationalise these intervention components. It is therefore imperative that those designing and delivering interventions to support long-term PA behaviour change provide appropriate and accessible explanations of the fundamental BCTs to ensure they can be enacted. Assessing this during and after intervention delivery is also important.

In addition to the need for evidence informed intervention components, it is important to understand *how* these components exert their effects on behaviour. This paper identifies potentially effective intervention components/BCTs linked to the constructs of the conceptual model and goes on to state the hypothesised MoA through which these BCTs/groups of BCTs exert their effect on behaviour. This was achieved by using a publicly available database where links between BCTs and proposed MoA have been collated based on expert-verified consensus [34]. It has been widely acknowledged that research exploring potential MoA as mediators of behaviour change in specific context with specific populations is necessary to advance the field of behavioural science. Haggar et al. [42] describe a process model, including the type of data and analysis needed to contribute to the evidence base of testing proposed MoA. Such contributions will provide data that can be synthesised and increase our collective knowledge of the MoA of interventions. This is important work given the current lack of such evidence, as demonstrated in a recent series of meta-reviews [43].

## Conclusion

This conceptual model and accompanying inventory of potential intervention components is intended to inform those developing novel interventions to promote long-term engagement in physical activity after a cancer diagnosis. The model and its proposed MoA could be tested in the future, using methods outlined by Haggar and colleagues. Use of this model alongside participatory approaches, consulting with end users and key stakeholders to ensure relevance and appropriateness, is encouraged.

## Data Availability

The data generated during the current study are available from the corresponding author on reasonable request.

## Abbreviations

PA: Physical activity
BCT: Behaviour change technique
TDF: Theoretical Domains Framework
SCT: Social cognitive theory
TTM: Transtheoretical model
MoA: Mechanism of action

## Declarations

### Disclaimer

The views expressed are those of the authors and not necessarily those of the NHS, the NIHR or the Department of Health and Social Care.
